# Theoretical, chemical, and electrochemical studies of *Equisetum arvense* extract as an impactful inhibitor of steel corrosion in 2 M HCl electrolyte

**DOI:** 10.1038/s41598-022-06215-6

**Published:** 2022-02-10

**Authors:** M. A. Deyab, Q. Mohsen, Lei Guo

**Affiliations:** 1 Egyptian Petroleum Research Institute (EPRI), Nasr City, Cairo, Egypt; 2 Department of Chemistry, College of Sciences, Taif University, Taif, Saudi Arabia; 3 School of Material and Chemical Engineering, Tongren University, Tongren, 554300 China

**Keywords:** Chemistry, Electrochemistry

## Abstract

A new type of *Equisetum arvense* aerial part (EAAP) extract was ready to be tested as a corrosion inhibitor for steel-based parts in Multi-stage flash (MSF) segments while the segments were being acid cleaned. The EAAP extract was identified using Fourier-transform infrared (FTIR) and High-performance liquid chromatography (HPLC). When compared to the specimen exposed to blank solution, EAAP extract molecules covered about 97% of the carbon steel surface in 2 M HCl solution, and the corrosion rate was reduced to 0.58 ± 0.02 μg cm^−2^ h^−1^ at 300 mg l^−1^. EAAP extract tends to have a blended impact on both anodic and cathodic sites on the surface of carbon steel. The thermodynamic activation factors are substantially higher in the presence of extract solution than in the absent of extract, demonstrating that the carbon steel surface would corrode slowly in the presence of EAAP extract. Theoretical models were used to validate the adsorption of EAAP extract on steel surfaces.

## Introduction

The desalination of seawater is among the alternatives to significantly boost global water availability^1^. The Multistage Flash Procedure (MSF) represents a significant proportion of globally desalination plants. MSF has various segments made of different steel–based materials and alloys^2^. Scaling is one of the major operations and maintenance problems that affect the effectiveness of MSF production^3–5^. Acid cleaning products have been used in practice for removing scales layers from surfaces of the metal structures. In the pickling solution, hydrochloric acid is usually employed^5^. The result is high corrosion damage to the surface of the metals. Corrosion inhibitors are the primary strategy for controlling corrosion in steel-based materials in the MSF segments^6–8^. In particular, organic materials possess hetero-atoms (i.e. O, N, S) in their molecules have been discovered as acid cleaning inhibitors^9–12^. Even though these inhibitors have performed successfully, many are costly and hazardous to people and the environment.

The use of extracts as safe and low-cost corrosion inhibitors is a significant development in corrosion research. Many research works of various section of the plant have been confirmed for corrosion-inhibiting applications, such as flower, leaf, root or even whole plant^13–18^. Various plant sections contain various phytochemicals concentrations and types as reported by Umoren et al.^19^. Tunbergia fragrans inhibited mild steel corrosion in 1 M HCl, according to Muthukumarasamy et al.^20^. At 500 ppm concentration, the extract had a maximum efficiency of 81%. Because plant extracts have chemical structures that are close with those of organic conventional organic molecules, they can play a role as corrosion inhibitors. Alibakhshi et al.^21^ used infrared spectroscopy to examine the molecular structure of Persian liquorice leaves. They found that the presence of OH, COOH, and CO functional groups in Persian liquorice leaf extracts may help to prevent corrosion by coordinating with Fe atoms on the steel surface. Ramrez-Peralta et al.^22^ evaluated the impact of Equisetum arvense extract to protect the steel from corrosion in 0.5 M sulfuric acid. The maceration procedure of leaves and stems was used to create this extract.

The present study objective is to generate particularly successful corrosion inhibitors for steel-based materials in the MSF segments that are also low in toxicity. Through this view, the *Equisetum arvense* aerial part (EAAP) extract was explored during acid cleaning as an inhibitor of corrosion of steel-based materials. We used multi-solvent extraction at high temperatures to extract more phyto-chemicals from the EAAP plant in this study. In addition, we employed theoretical concepts to confirm the efficacy of EAAP extract as a corrosion inhibitor.

## Materials and methods

### Materials

An Egyptian thermal water treatment plant provided the carbon steel (0.23 C, 0.035 P, 0.007 Si, 0.34 Mn, and the overall balance Fe). The acid cleanser is a 2.0 M HCl solution from Sigma Aldrich. Herbal family group (Egypt) supplied Equisetum arvense aerial part (EAAP) powder. 10 g of EAAP powder were weighted and blended with 100 ml of stock solution (8% ethyl acetate (purity > 99%) + 22% distilled H_2_O + 70% C_2_H_5_OH (purity 99.8%) in a beaker. For five hours, the mixture was embedded in a water pool set at 348 K, with regular mixing. The mixture had been then disconnected from the bath and allowed to cool for 72 h prior to actually being filtered by Lab vacuum filtration apparatus*.* The extract was vacuumed to 313 K till the solvents were completely eliminated. The basic constituents of the EAAP extract have been identified with the use of Liquid chromatography (Shimadzu CO.) and FTIR (Perkinelme spectrometer). For FTIR analysis, the sample was analyzed in pure phase in KBr plate and the spectra were taken in the 4000–400 cm^−1^ range.

### Corrosion rate calculations

The EAAP extract corrosion inhibition ability is evaluated through mass loss and electrochemical calculations. The flow velocity of the solution was scheduled at 2.0 m s^−1^ in aerated circumstances.

For mass loss examinations, carbon steel samples were carefully cut into sheets with dimensions of 1.0 cm × 1.5 cm × 0.05 cm. The mass loss process was implemented in accordance with ASTM G1-03(2017)e1 approach^23^. Carbon steel samples were polished with a variety of emery sheets varying from 800 to 2800 grades before each experiment. In 2.0 M HCl solution, the immersion period for carbon steel samples is 4 h. The following is used to determine the rate of corrosion (*C*_R_)^24^:

1 $$C_{R} = \frac{W}{A \times t}$$

*W* symbolizes mass loss (mg), *A* symbolizes carbon steel surface area (cm^2^), and *t* symbolizes immersion time (h). For accuracy, the test was repeated, and the statistics were averaged over three trials.

EAAP extract inhibitory activity efficiency (*η*_W_%) was obtained as follows^25^:

2 $$\eta_{\rm{W}} {\% } = \frac{{C_{{\rm{R0}}} - C_{\rm{R}} }}{{C_{{\rm{R0}}} }} \times {100}$$

For blank acidic medium, *C*_R0_ has been recorded.

The electrochemical testing was carried out using a potentiostat/galvanostat (Gamry-reference 3000). The laboratory electrochemical cell was already demonstrated by Deyab^26^. A standard calomel electrode (SCE), steel working electrode (surface area 0.564 cm^-2^) and Pt wire electrode are included within the experimental cell. The Tafel graph was built using a scan rate of 1.25 mV s^−1^. EAAP extract (*η*_j_%) inhibition efficacy was calculated using the formulation^27^:

3 $$\eta_{\rm{j}} {\% } = \frac{{j_{{\rm{corr(0)}}} - j_{{\rm{corr}}} }}{{j_{{\rm{corr(0)}}} }} \times {100}$$

where *j*_corr(0)_ and *j*_corr_ are the corrosion current densities measured in the absence of EAAP extract and in the presence of EAAP extract. Electrochemical impedance testing (EIS) process criteria include a 100 kHz to 0.01 Hz frequency range and 20 mV amplitude for OCP**.** The OCP of the working electrode is monitored over time before EIS and polarization studies, until it reaches a steady state value after 60 min. Gamry Echem Analyst software was used for EIS results extraction and fitting procedure.

### Computational study

Quantum chemical calculations dependent on the DFT model were performed for the four major ingredients, namely caftaric acid, kaempferol-3,7-di-*O*-glucoside, kaempferol-3-O-rutinoside, and isoquercetin, in order to investigate the reaction activities of as-prepared EAAP extract. Geometry optimization was carried out using the DMol^3^ program (BIOVIA Materials Studio) Numerous quantum chemical properties were recognized and obtained, such as *E*_HOMO_, *E*_LUMO_, Δ*E* (gap energy), *μ* (dipole moment), *χ* (electronegativity), and *η* (global hardness). The Forcite module and Materials Studio the package were used to explore the adsorption behavior of four major components on an iron surface. The calculations were carried out in a simulation box comprised of Fe(110) substrate and solvent layer (600 H_2_O + one ingredient molecule).

### Surface morphology

ZEISS scan electron microscopy (SEM) was used to examine the surface morphology of carbon steel after immersed in different solutions for 4.0 h at 298 K.

We confirm that all methods were performed in accordance with the relevant guidelines and regulations.

## Results and discussion

### The major constituents of EAAP extract

**Figure 1:**
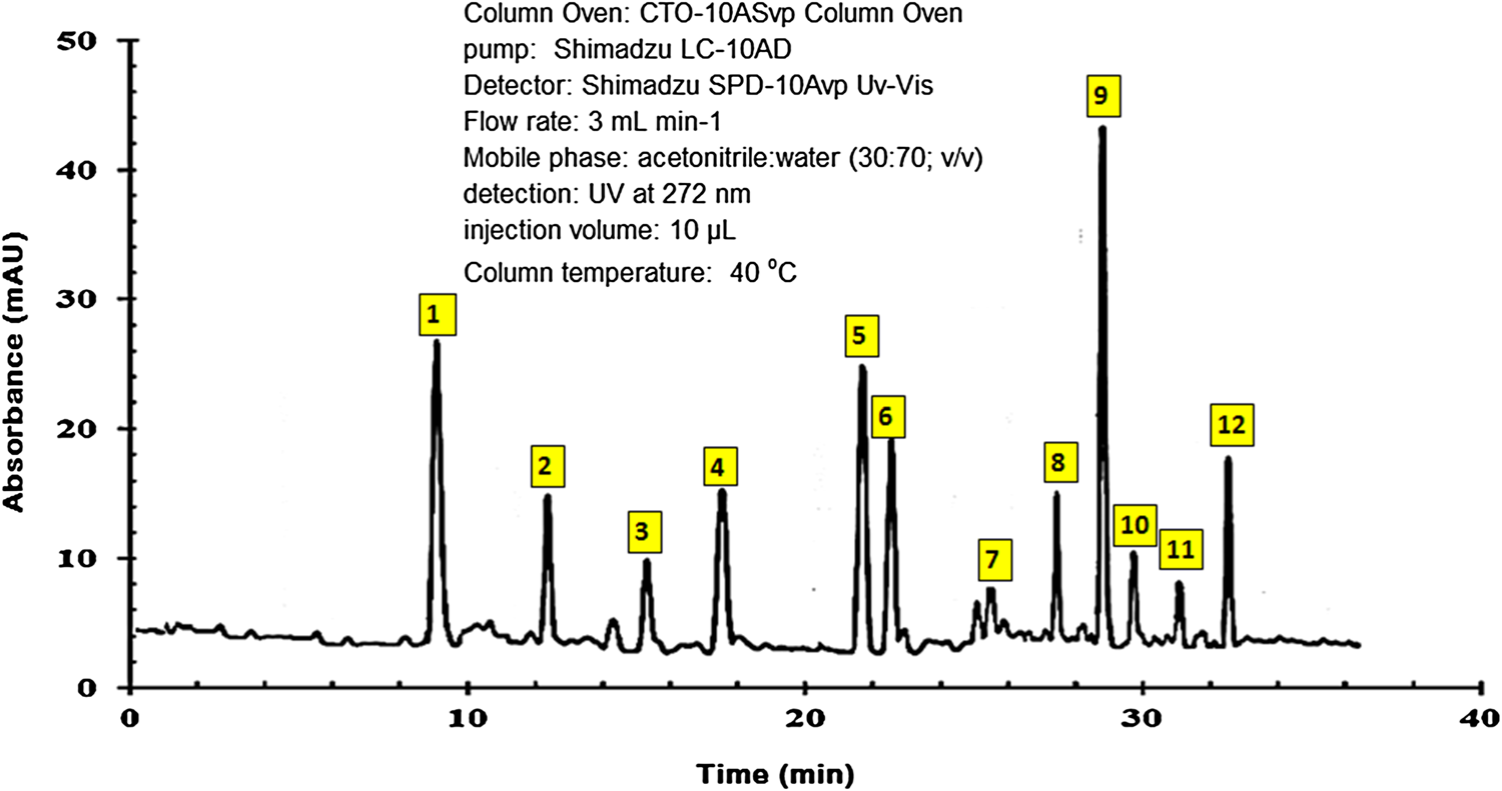
HPLC chromatogram of EAAP extract.

**Figure 2:**
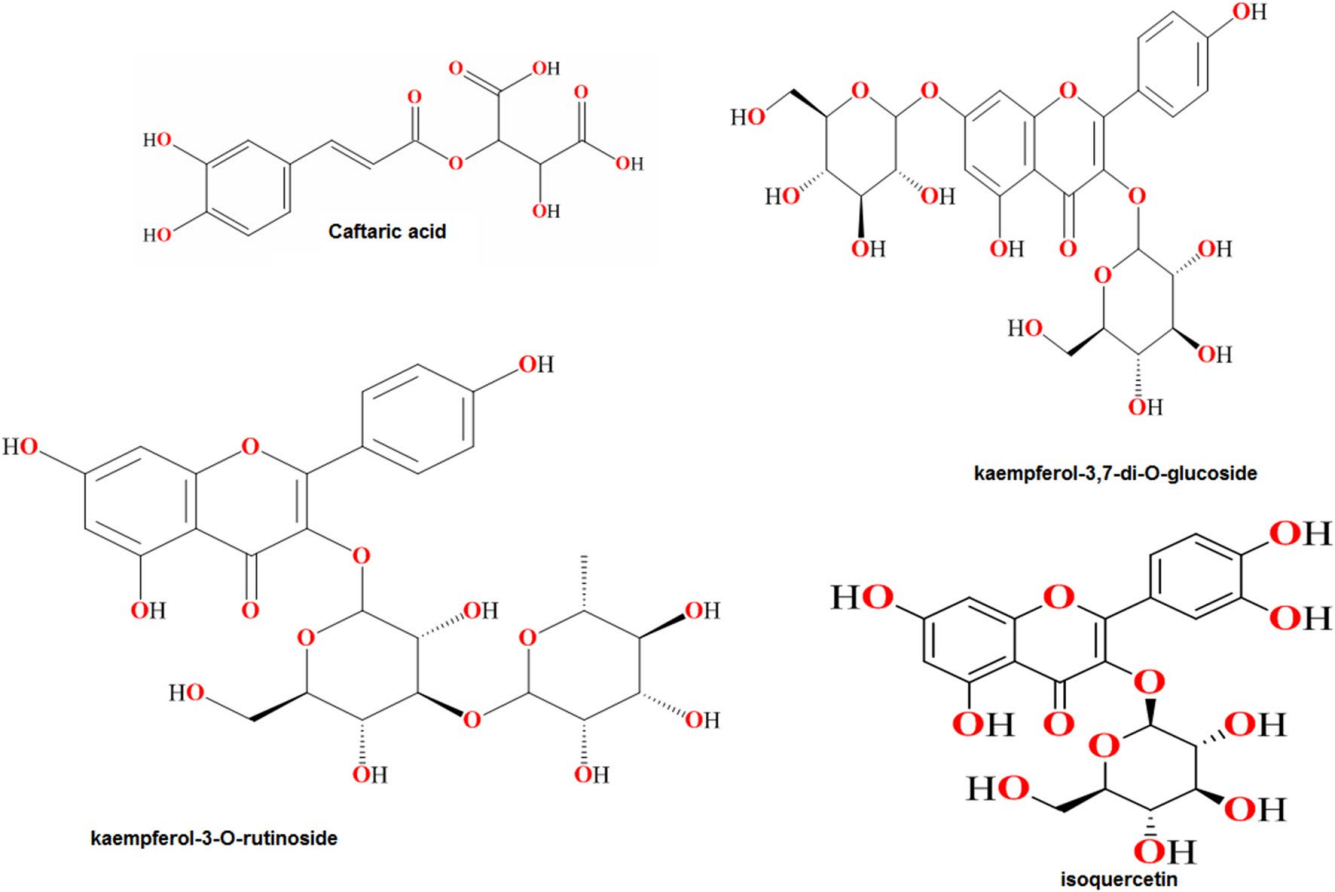
the molecular structures of major ingredients in EAAP extract.

The most important components of EAAP extract were determined by HPLC inspection^28^, as seen in Fig. 1. The retention times, chemicals formulas and names are reported in Table 1. Caftaric acid, kaempferol-3,7-di-*O*-glucoside, kaempferol-3-*O*-rutinoside, and isoquercetin were identified as the primary peaks 1, 5, 6, and 9, respectively. Figure 2 depicts the molecular structures of these compounds.

**Table 1:** The main chemical components of EAAP extract.

Peak no.	Retention time (min)	Chemical formula	Compound name
1	8.7	C_13_H_12_O_9_	Caftaric acid
2	12.2	C_33_H_40_O_21_	Kaempferol 3-sophoroside-7-glucoside
3	15.7	C_27_H_30_O_17_	Quercetin-3,7-di-*O*-glucoside
4	17.1	C_13_H_12_O_8_	Caffeoyl-malic acid
5	21.2	C_27_H_30_O_16_	Kaempferol-3,7-di-*O*-glucoside
6	22.6	C_27_H_30_O_15_	Kaempferol-3-*O*-rutinoside
7	25.8	C_9_H_8_O_3_	Coumaric acid
8	26.9	C_10_H_10_O_4_	Methyl caffeate
9	28.7	C_21_H_20_O_12_	Isoquercetin
10	29.4	C_21_H_20_O_10_	Apigenin-*O*-glucoside
11	30.8	C_21_H_20_O_11_	Astragalin
12	32.5	C_22_H_18_O_12_	Cichoric acid

**Figure 3:**
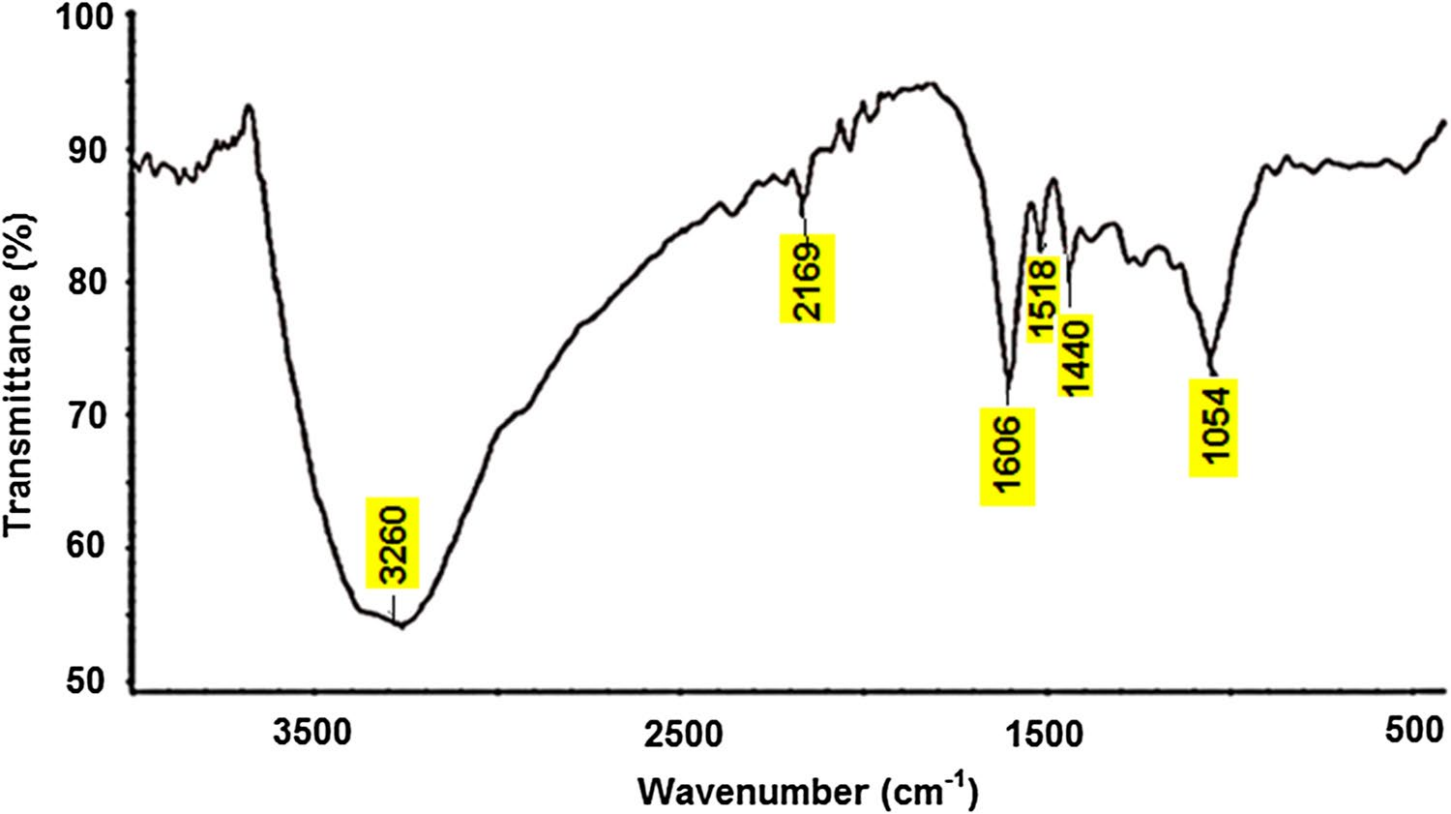
FT-IR spectrum of EAAP extract.

The data from FT-IR spectral analysis of EAAP extract, as shown in Fig. 3, revealed the presence of different functional groups^29^. The broad band at 3260 cm^−1^ is assigned to O–H stretching in the COOH group. Due to aliphatic CH_2_, the band has asymmetric and symmetrical vibration stretches at 2169 cm^−1^. The band at 1606 cm^−1^ is due to the CO in CHO group. Bands at 1518 and 1440 cm^−1^ are due to C=O stretching and C–H deformation, respectively.

### Corrosion inhibition measurements

The outcomes of the testing mass loss work are shown in Table 2. In comparison to the blank solution (2 M HCl solution), the corrosion rate of carbon steel suspended in the acid cleaning solution in the presence of EAAP extract is relatively small, as shown in Table 2. The efficiency of EAAP extract (*η*_*W*_%) reaches its maximum a high level at 300 mg l^−1^ (95.2%). Above this concentration (i.e. > 300 mg l^−1^), there is no noticeable change in *η*_*W*_%.

**Table 2:** Mass loss analysis for carbon steel in 2 M HCl solution in the absence and presence of EAAP extract at 298 K.

EAAP extract (mg l^−1^)	*C*_R_ (μg cm^−2^ h^−1^)	*η*_W_%
Blank	12.23 ± 0.33	–
25	9.24 ± 0.42	24. 4
50	6.38 ± 0.24	47.8
100	1.01 ± 0.04	91.7
200	0.62 ± 0.02	94.9
300	0.58 ± 0.02	95.2

**Figure 4:**
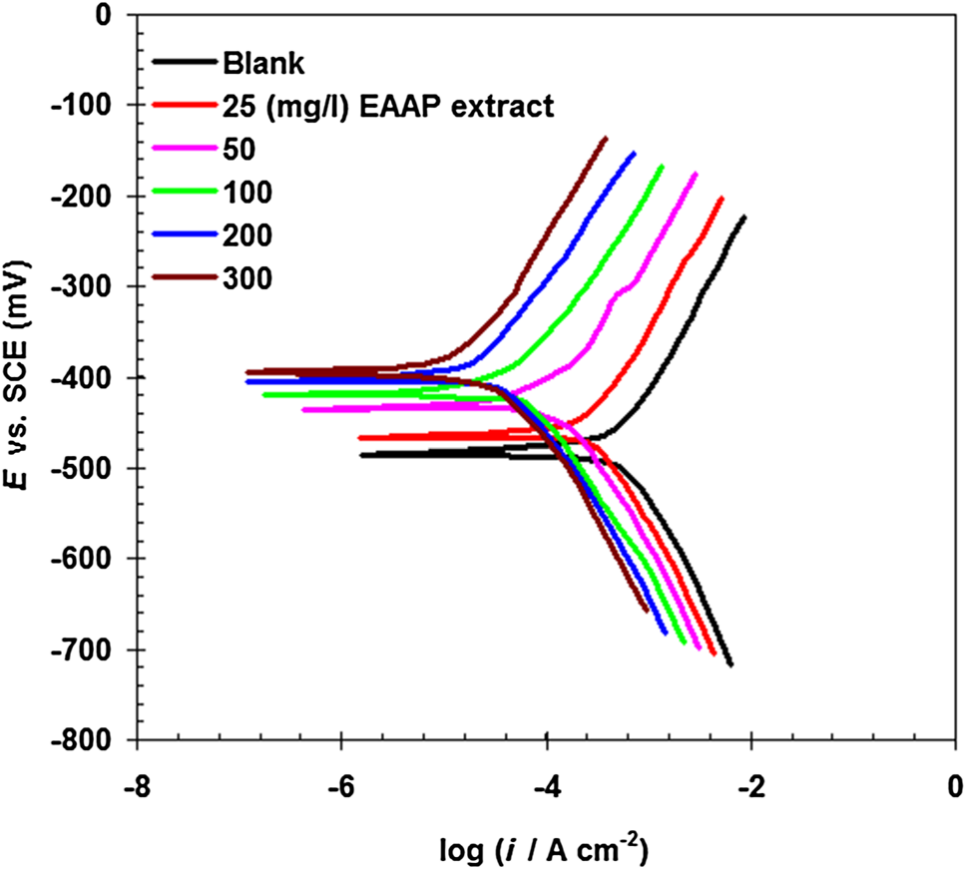
Potentiodynamic polarization curves of carbon steel in 2 M HCl solution in the absence and presence of EAAP extract at 298 K and scan rate of 1.25 mV s^−1^ (immersion time 60 min).

Polarizations provide critical information in acid solution containing EAAP extract on carbon steel corrosion processes. Curves of carbon polarization in 2 M HCl solution are illustrated in Fig. 4 in relation to EAAP extract concentrations. Table 3 summarizes the polarization parameters. EAAP extract has a highest shift of approximately 89 mV in the corrosion potential (*E*_corr_) in comparison to *E*_corr_ in a blank solution. This indicates that EAAP extract seems to have a mixed impact on the surface of carbon steel and occupies simultaneously anodic and cathodic places with a significant anode action^30,31^. The *j*_corr_ value declines with rising EAAP extract content until it reaches 300 mg l^−1^. Clearly, when compared to published studies for various similar plants extract inhibitors, the maximum inhibition result (97.4%) obtained at 300 mg l^−1^ of EAAP extract (Table 3) may be considerable. For instance, Primula vulgaris extract has a significant impact (efficiency 95.5%) on steel corrosion in 1.0 M HCl solution, according to Tabatabaei Majd et al.^32^. Shahmoradi et al.^33^ investigated the corrosion prevention property of Juglone in mild steel 1 M HCl. After 8 h, an efficiency of 95% was reached utilising 800 ppm Juglone. Guruprasad and Sachin^34^ investigated the corrosion protection ability of Amorphophallus paeoniifolius leaves APL extract on steel surface in 1 M HCl solution. At a 10% v/v dosage of APL extract, the corrosion inhibition efficiency was 92.4%.

**Table 3:** Polarization parameters for carbon steel in 2 M HCl solution in the absence and presence of EAAP extract at 298 K.

EAAP extract (mg l^−1^)	*E*_corr_, mV (SCE)	*j*_corr_ (μA cm^−2^)	*η*_j_%
Blank	−486	511.5	–
25	−462	329.4	35. 6
50	−434	116.1	77.3
100	−420	34.7	93.2
200	−407	18.4	96.4
300	−397	13.2	97.4

**Figure 5:**
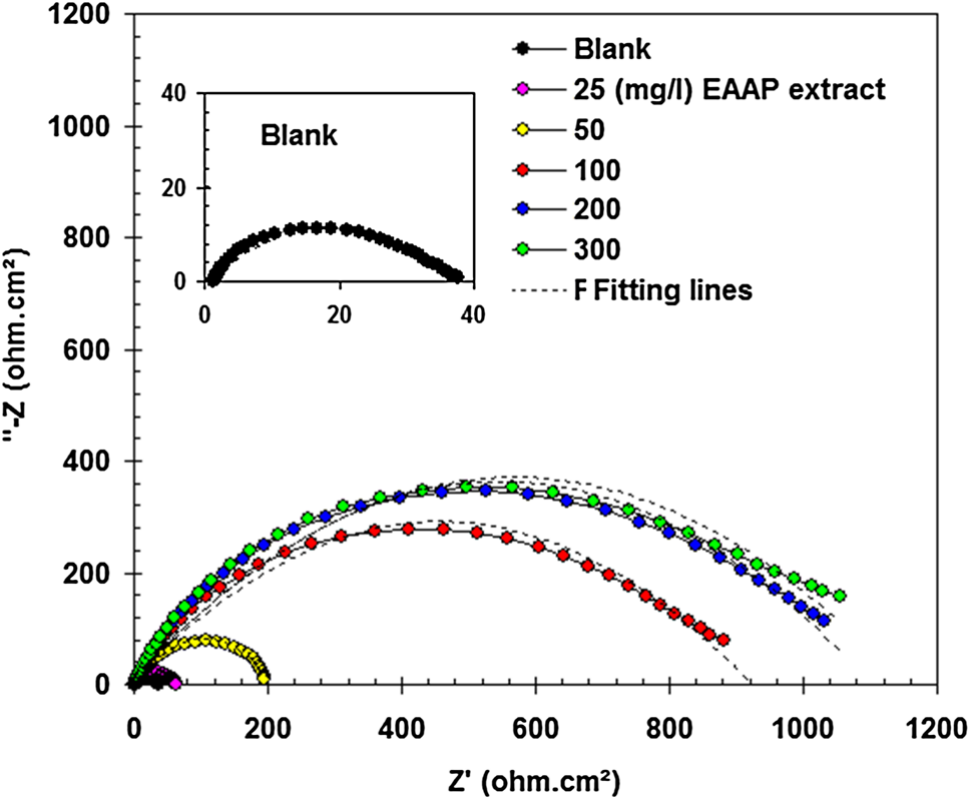
Nyquist plots of carbon steel in 2 M HCl solution in the absence and presence of EAAP extract at 298 K (immersion time 60 min).

**Figure 6:**
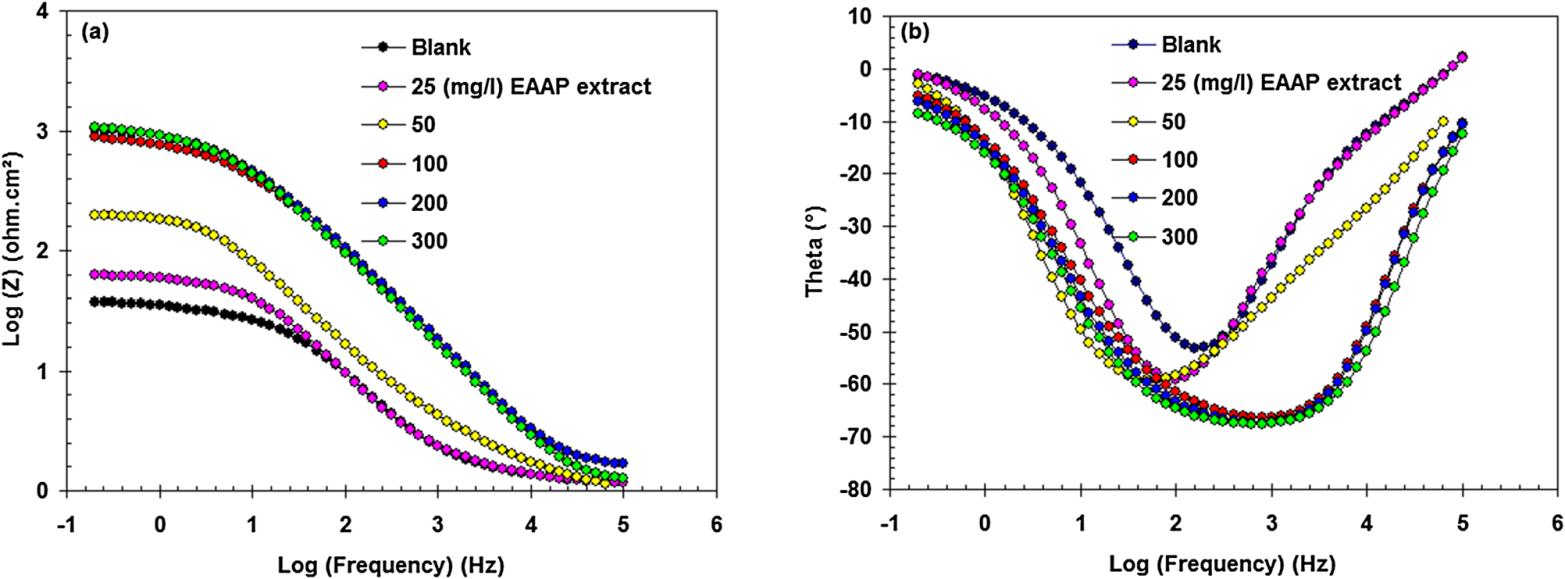
Bode-module **(a)** and Bode-phase angle **(b)** plots of carbon steel in 2 M HCl solution in the absence and presence of EAAP extract at 298 K.

**Figure 7:**
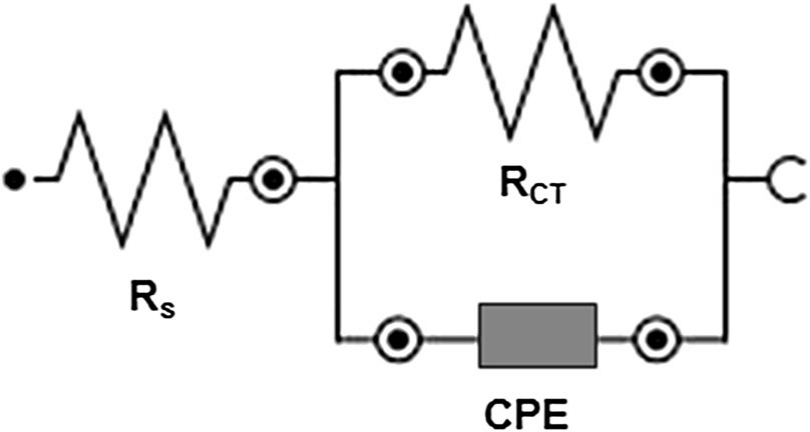
The equivalent circuit for fitting of the impedance data.

EIS is particularly useful as a non-destructive approach for assessing corrosion inhibition actions^35^. In the absence and presence of EAAP extract, the EIS response (Nyquist and Bode) of carbon steel in 2 M HCl solution is reported in Figs. 5 and 6. The large capacitive loop in Fig. 5 represents the adsorption of the EAAP extract molecules (active chemicals) on the carbon steel samples^36,37^. This capacitive loop is caused by charge transfer resistance (R_CT_) and electric double layer capacitance (C_dl_)^38^. The appearance of a single semicircle revealed that the single charge transfer procedure is not influenced by inhibiting molecules during dissolution. Constant phase element (CPE) was used instead of real capacitance since the resulting plots showed depressed semicircles^39,40^. As can be shown in Table 4, with EAAP extract concentration, the R_CT_ levels of inhibited compounds rose. In addition, the CPE values declined (Table 4). This is because the local dielectric constant declines and/or the electrical dual layer thickness increases via metal-solution-interface extract adsorption^25,41^. For best capacitance performance, the slope of the Bode-module would be 1 and the Bode-phase angle would be -90° in the middle frequency zone^42^. The slopes and phase angles (Fig. 6) reach a higher steady state and their ranges are nearer to 1 and 90°, respectively, in the addition of EAAP extract than in the absence, demonstrating the inhibitory activity of the EAAP extract on the carbon steel surface. The corresponding circuit design utilized to meet the experimental EIS finding is depicted in Fig. 7. The variables of charge transfer resistance in blank (R_CT0_) and inhibited solution (R_CT_) can be used to assess the performance of EAAP extract as corrosion inhibitor (*η*_R_%) using the supplied formula^43^.

4 $$\eta_{\rm{R}} \% = \frac{{R_{{\rm{CT}}} - R_{{\rm{CTo}}} }}{{R_{{\rm{CT}}} }} \times 100$$

**Table 4:** EIS parameters for carbon steel in 2 M HCl solution in the absence and presence of EAAP extract at 298 K.

EAAP extract (mg l^−1^)	R_CT_ (ohm cm^2^)	CPE (µF cm^−2^)	*η*_R_%
Blank	35.7	725.4	–
25	60.5	454.7	40.9
50	192.4	276.4	81.4
100	850.4	198.3	95.7
200	1024.8	105.2	96.5
300	1054.9	89.9	96.6

When the EAAP extract content was extended to 300 mg l^−1^, the *η*_R_% value was equivalent to 96.6% (Table 4), showing that the EAAP extract had excellent anticorrosion characteristics. In all, the mass loss, EIS and polarization experiments indicated that the efficiency factors of inhibition of corrosion in the various procedures were almost equal.

### Aspects for kinetics and adsorption

Further research has been conducted to examine the correlation between steel corrosion and increase in temperature (from 298 to 328 K) on EAAP's efficiency extract (Table 5). The findings show that the rate of corrosion of steel in acid solution (both inhibited and uninhibited) tends to accelerate as temperature rises. The inhibition efficiency gradually decreases as temperature rises (Table 5), implying a physisorption mode^44^. Even though the difference was not significant, it indicates that the extract/surface system is stable at elevated temperature.

**Table 5:** Mass loss for carbon steel in 2 M HCl solution in the presence/absence of EAAP extracts (300 mg l^−1^) at different temperatures.

Temperature (K)	EAAP	*C*_*R*_ (μg cm^−2^ h^−1^)	*η*_*W*_ (%)
298	0	12.23 ± 0.33	−
	+	0.58 ± 0.02	95.2
308	0	15.23 ± 0.41	−
	+	0.98 ± 0.12	93.5
318	0	17.54 ± 0.47	−
	+	1.45 ± 0.15	91.7
328	0	21.54 ± 0.50	−
	+	2.30 ± 0.19	89.3

**Figure 8:**
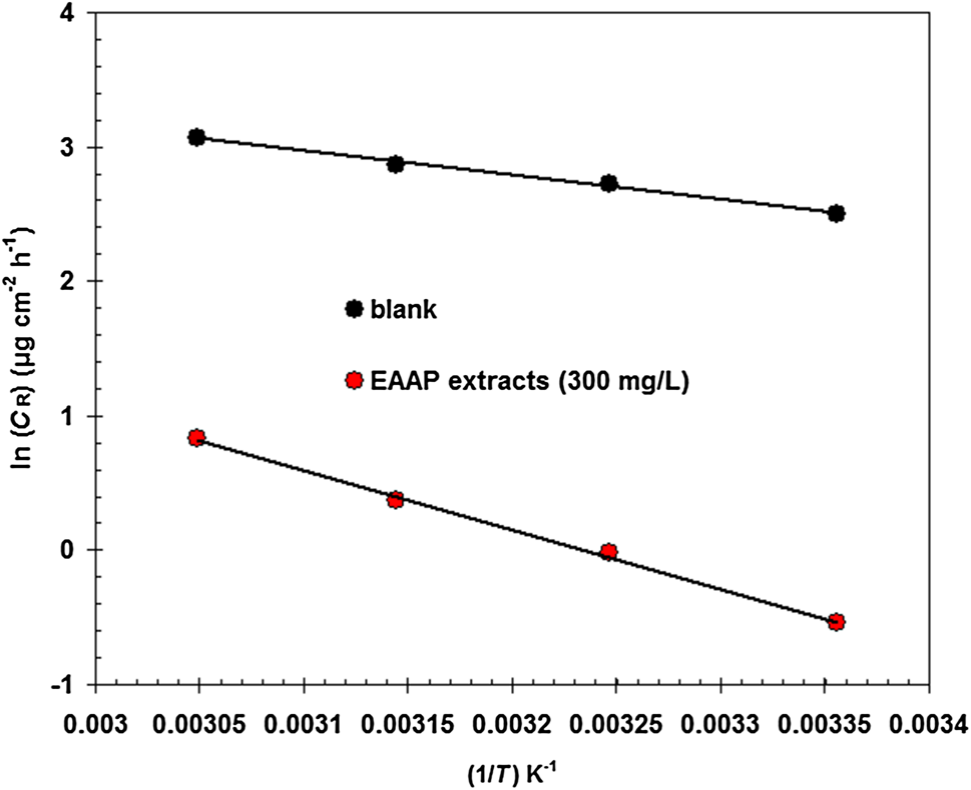
Comparison of Arrhenius plots for carbon steel in 2 M HCl solution in the presence/absence of EAAP extracts (300 mg l^−1^).

The Arrhenius formula (Eq. 5) describes how the corrosion rate (*C*_R_) of carbon steel in 2 M HCl solution in the presence/absence of EAAP extracts (300 mg l^−1^) varies with temperature^45^.

5 $$C_{R} = A\exp (\frac{{ - E_{a} }}{RT})$$

where *E*_a_ symbolizes activation energy, *T* symbolizes absolute temperature, *R* symbolizes molar gas constant and *A* symbolizes frequency factor. The corresponding Arrhenius plots are shown in Fig. 8. Without the EAAP extract, the corrosion oxidation of carbon steel in 2 M HCl solution appeared to have an *E*_a_ of nearly 14.9 kJ mol^−1^. For 300 mg l^−1^ of EAAP extract, *E*_a_ was altered to 36.7 kJ mol^−1^. The EAAP extract solution showed a higher *E*_a_ value than the blank solution, reflecting that a physical adsorption process occurred^46^.

The thermodynamic activation values for carbon steel in 2 M HCl solution, such as change in enthalpy (ΔH*) and entropy change (ΔS*), were deduced using the transition state equation (Eq. 6)^47^.

6 $$C_{R} = \frac{RT}{{Nh}}\exp (\frac{{\Delta S^{ * } }}{R})\exp (\frac{{ - \Delta H^{*} }}{RT})$$

where *N* means Avogadro's number and *h* means Planck's constant.

**Figure 9:**
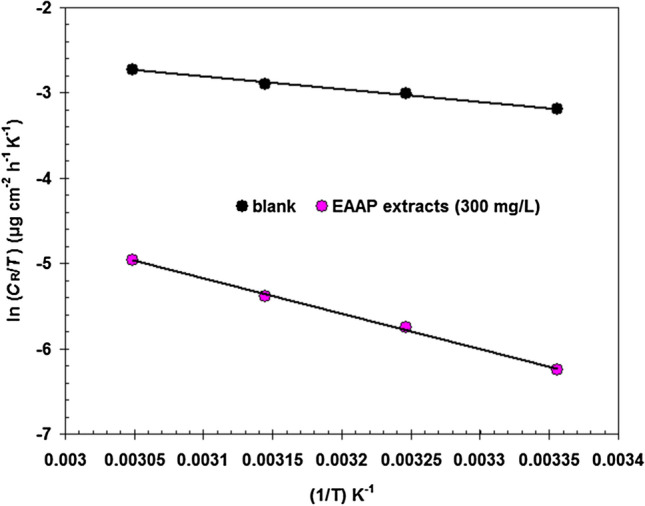
Comparison of transition state plots for carbon steel in 2 M HCl solution in the presence/absence of EAAP extracts (300 mg l^−1^).

The corresponding transition state plots are shown in Fig. 9.

The ΔH* value for carbon steel dissolving in 2 M HCl solution containing 300 mg l^−1^ of EAAP extract is substantially higher (34.15 kJ mol^−1^) than in the blank acid solution (12.32 kJ mol^−1^) suggesting a slow corrosion rate of the carbon steel surface in the presence of EAAP extract. Additionally, ΔH* exhibits a positive sign, suggesting that the carbon steel dissolving process is endothermic in 2 M HCl^48^. By comparing the ΔS* values of inhibited and blank acid solutions, the inhibited system (−224.8 J mol^−1^ K^−1^) exhibits a lower ΔS* value than the blank system (−153.2 J mol^−1^ K^−1^). This is primarily likely due to the reduction in disorder that happened after the reactants were transformed to the activated complex by the addition of EAAP extract^49^.

To validate adsorption for this process, the Langmuir isotherm model (Eq. 7) is utilized^50^.

7 $$\frac{{C_{{\rm{inh}}} }}{\theta } = \frac{1}{{K_{\begin{subarray}{l} \rm{ads} \\ \end{subarray} } }} + C_{{\rm{inh}}}$$

(*θ* = *η*_*W*_%/100^51^ = surface coverage, *C*_inh_ = EAAP extract concentration; *K*_ads_ = equilibrium constant).

**Figure 10:**
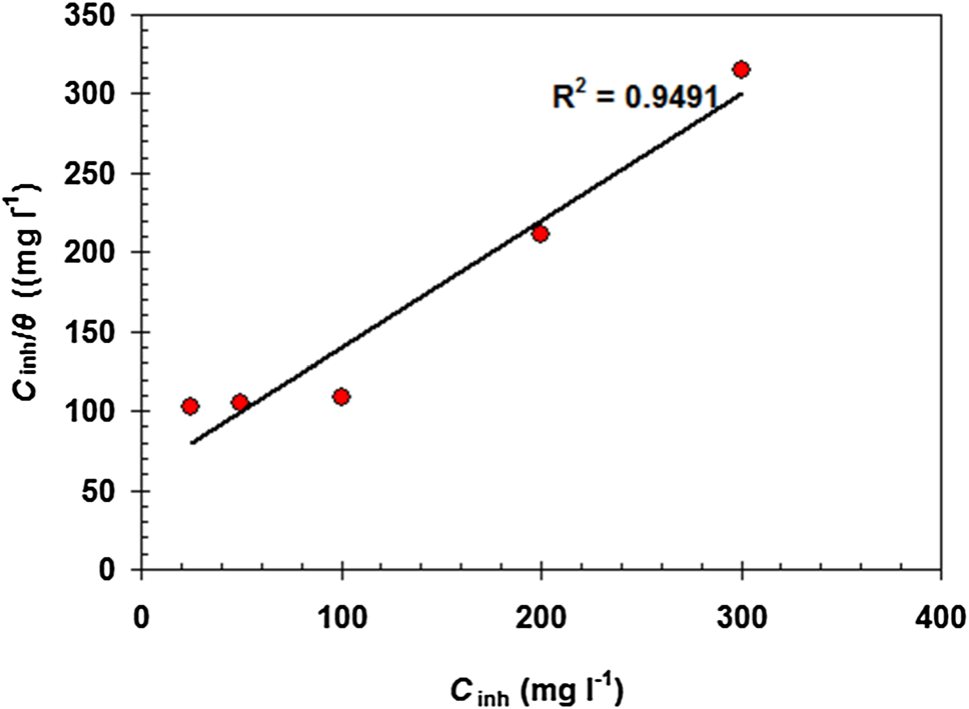
Langmuir adsorption isotherm for EAAP extract on the carbon steel at 298 K.

The Langmuir isotherm for EAAP extract is depicted in Fig. 10. The value of the correlation coefficients (R^2^) in Fig. 10 is considerably closer to one (i.e. 0.9491), showing that this procedure is valid in defining the adsorption behavior^52^. Furthermore, the EAAP extract's physical adsorption characteristics are demonstrated by the smallest *K*_ads_ value (0.0166 l mg^-1^)^53^.

### Theoretical considerations

#### DFT calculations

**Figure 11:**
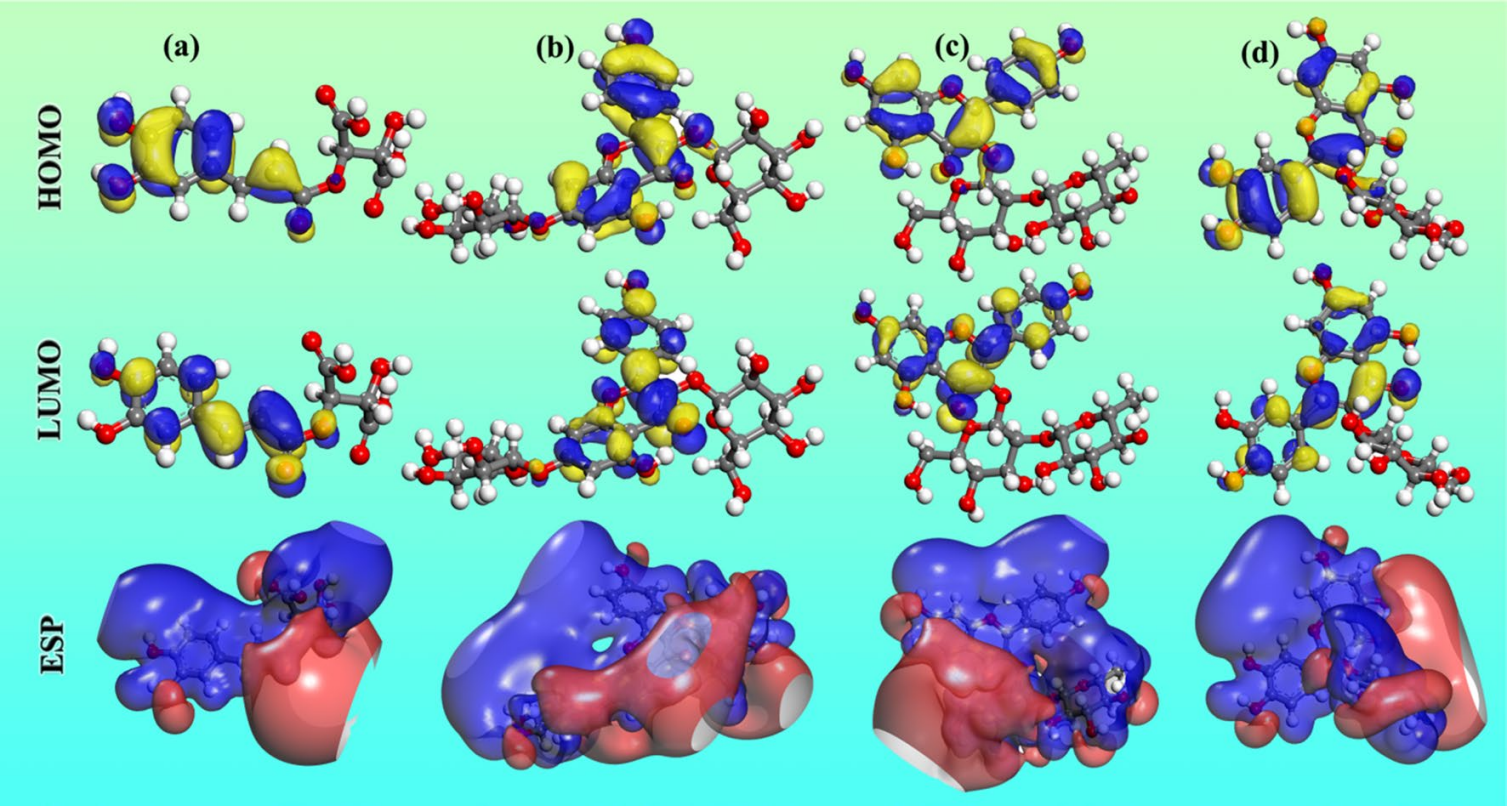
HOMOs, LUMOs, and ESP distributions for the investigated components: **(a)** caftaric acid, **(b)** kaempferol-3,7-di-*O*-glucoside, **(c)** kaempferol-3-*O*-rutinoside, and **(d)** isoquercetin.

To recognize the molecular function of the ingredients under consideration, quantum chemical calculations dependent on the DFT model were performed. As a result, the accompanying chemical properties of four components are depicted in Fig. 11. Typically, the HOMO orbital represents the molecule's electron-donating capacity, while the LUMO orbital represents the molecule's electron-acquiring capacity. The electron cloud of HOMO and LUMO for caftaric acid is clearly found to be almost located at the aromatic ring group. Other ingredients have an analogy to this situation. This demonstrates that these active adsorption sites can share electrons with metals in order to forming covalent bonds. Furthermore, the electrostatic potential (ESP) plots are divided into red and blue territories that represent nucleophilic and electrophilic nature, respectively. The red area is mostly concentrated around the hydroxyl groups. This implies that the red areas are also the major active adsorption sites when adsorbing at the steel substrate.

Table 6 lists some relevant descriptors. The *μ* values of caftaric acid, kaempferol-3,7-di-*O*-glucoside, kaempferol-3-*O*-rutinoside, and isoquercetin are 11.72, 12.41, 12.18, and 11.10 Debye, respectively. In general, a high dipole moment increases adsorption on the metal surface and thus the inhibition effectiveness^54^. Therefore, the anti-corrosion ability of kaempferol-3,7-di-*O*-glucoside and kaempferol-3-*O*-rutinoside are better than the other two ingredients, which can be substantiated by their lower Δ*E* values. The fraction of electrons (Δ*N*) transferred between both the adsorbate molecules and the metal surface is calculated using the equation below^55^:

8 $$\Delta N = \frac{{\Phi_{{{\text{Fe}}}} - \chi_{{{\text{inh}}}} }}{{2(\eta_{{{\text{Fe}}}} + \eta_{{{\text{inh}}}} )}}$$

where in a theoretical value of work-function *Φ*_Fe_ = 4.82 eV was used for the iron surface, *η*_inh_ represents the hardness of inhibitor, *η*_Fe_ was set to 0 assuming *I* = *A* for the bulk metal. When *N* is greater than zero, the inhibitor molecule transfers its own electrons to metal, and vice versa when *N* is less than zero. The all positive Δ*N* values shown in Table 6 suggest that EAAP extract has the ability to donate electrons to carbon steel surface.

**Table 6:** Quantum chemical descriptors for primary ingredients of EAAP extract at GGA/BLYP/COSMO level.

Component	*E*_HOMO_ (eV)	*E*_LUMO_ (eV)	Δ*E* (eV)	*I* (eV)	*A* (eV)	*μ* (Debye)	*χ* (eV)	*η* (eV)	Δ*N*
Caftaric acid	− 5.321	− 2.902	2.419	5.321	2.902	11.72	4.111	1.209	0.292
Kaempferol-3,7-di-*O*-glucoside	− 5.351	− 2.772	2.579	5.351	2.772	12.41	4.061	1.289	0.294
Kaempferol-3-*O*-rutinoside	− 5.208	− 2.658	2.550	5.208	2.658	12.18	3.933	1.275	0.347
Isoquercetin	− 5.205	− 2.705	2.500	5.205	2.705	11.10	3.955	1.250	0.346

#### Molecular dynamics simulation

**Figure 12:**
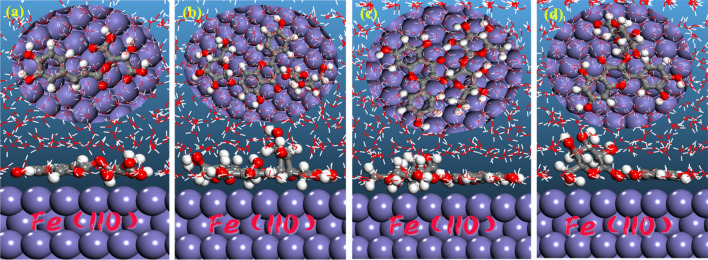
Side and top views of most stable adsorption configurations for four components on Fe(110) surface. **(a)** Caftaric acid, **(b)** kaempferol-3,7-di-*O*-glucoside, **(c)** kaempferol-3-*O*-rutinoside, and **(d)** isoquercetin.

The dynamics process was completed, and the entire system reached equilibrium when both the temperature and energy of the system were balanced. The low energy adsorption configurations of four ingredients adsorbed onto Fe(110) surface are shown in Fig. 12. The inhibitor molecules are adsorbed nearly flat on the metal substrate to optimise surface coverage and contact, order to ensure a powerful contact for the adsorbate/substrate state.

The adsorption energy (*E*_ads_) of corrosion inhibitors can be used to calculate the power of the corrosion inhibitors^56^:

9 $$E_{{{\text{ads}}}} = E_{{{\text{total}}}} - (E_{{\text{surf + water}}} + E_{{\text{inh + water}}} ) + E_{{{\text{water}}}}$$

(*E*_total_ is the energy of the entire system, *E*_surf+water_ is the total energy of Fe(110) surface and solution without the inhibitor, *E*_inh+water_ is the total energy of the inhibitor and solution; *E*_water_ is the potential energy of the solvent molecules).

The adsorption energies in this work were calculated from the average adsorption energy of the obtained equilibrium configurations. The obtained *E*_ads_ values are − 637.1, − 1107.3, − 1162.9, and − 810.6 kJ/mol for caftaric acid, kaempferol-3,7-di-*O*-glucoside, kaempferol-3-*O*-rutinoside, and isoquercetin, respectively. We can see that the adsorption energies are negative and thus spontaneous adsorption canbe expected. Generally, the bigger the absolute value of *E*_ads_ is, the stronger the interaction between the inhibitor and metal surface will be. Obviously, it appears that kaempferol-3,7-di-*O*-glucoside and kaempferol-3-*O*-rutinoside have higher absolute values of *E*_ads_ than caftaric acid and isoquercetin, therefore they may play the central role in the corrosion inhibition process.

### Mechanism of anti-corrosion properties of EAAP extract

**Figure 13:**
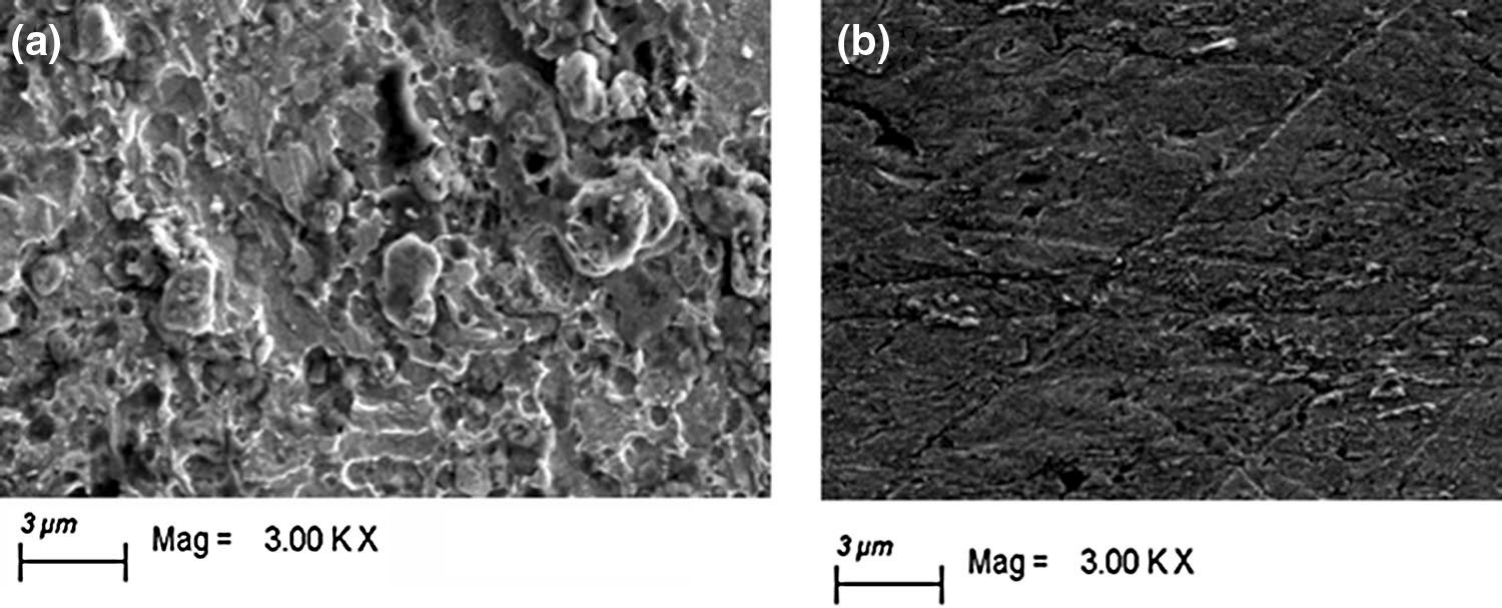
SEM images of the uninhibited **(a)**, and inhibited **(b)** carbon steel surface taken after 4 h exposure to 2 M HCl solution containing 300 mg l^−1^ EAAP at 298 K.

In general, most plant extract includes a variety of phyto-chemicals ranging in molecular structure from medium to complicated, with a variety of potential active (adsorption) centers in the combination of widespread cross linking between different functional groups and hetero-atomic multiple bonding^57–59^. Most such electron-rich hydrophilic centres connect directly (adsorb) with electrode surface, whereas hydrophobic phytochemicals keep flying in electrolyte and avoid touch with the metal substrate^60^. Because EAAP extract contains a variety of chemicals (Fig. 2), it is useful as a corrosion inhibitor. These compounds include a number of polar functional groups as well as many bonds. These electron-rich regions facilitate compound adsorption on the carbon steel surface^61–63^. Adsorption of EAAP extract molecules can be place by physisorption, chemisorption, or a combination of the two (physio-chemi-sorption). It is critical to understand that in an acidic solution, heteroatoms of EAAP extract molecules begin protonation and are moved to their protonated form^64^. The buildup of counter ions in the acidic solution results in the formation of a negative charge on the carbon steel surface^65^. Electrostatic attraction first coupled EAAP extract chemicals and carbon steel surfaces together. The cationic state of EAAP extract molecules, on the other hand, may be converted to their neutral state by receiving the electrons produced at the carbon steel surface. Upon becoming adsorbed, the EAAP extract molecules create a barrier at the interface between the acid electrolyte and the carbon steel surface^66^. The anti-corrosion impact and barrier protection efficiency of EAAP extract for carbon steel substrates in 2 M HCl solution was additionally verified by surface preservation and a decrease in the amount of corrosion products, as evidenced in SEM images in Fig. 13. The carbon steel substrate in blank liquid is severely destroyed, as seen in Fig. 13ba. In comparison, the substrate submerged in 300 mg l^−1^ EAAP-inhibited liquid has a substantially better surface with less corrosion products (Fig. 13b).

Table 7 reports that EAAP extract has a better inhibition efficacy for steel than other extract inhibitors in 2 M HCl solution^67–73^.

**Table 7:** Comparison of EAAP extract results with other extract inhibitors for steel in 2 M HCl.

Extract	Optimum con. of extract	Corrosive solutions	Maximum efficiency, %	References
*Aizoon canariense*	250 ppm	2 M HCl	82.6	^67^
Olive leaf extract	900 ppm	2 M HCl	91.0	^68^
*Conyza bonariensis*	100 ppm	2 M HCl	93.3	^69^
*Rosmarinus officinalis*	200 ppm	2 M HCl	89.18	^70^
Eggplant Peel	1000 ppm	2 M HCl	84.0	^71^
*Baphia nitida* leaves	100 ppm	2 M HCl	93.0	^72^
*Tectona grandis* leaf	1000 ppm	2 M HCl	71.7	^73^
*Equisetum arvense*	300 ppm	2 M HCl	97.4	This work

## Conclusions

Chemical and electrochemical examinations, as well as quantum chemical findings, indicated that the EAAP extract can be utilized as a corrosion inhibitor for steel-based components in the MSF segments during acid cleaning using a 2 M HCl solution. Polarization experiments revealed that the inhibitory effectiveness of EAAP extract was maximum (97.4%) in 2 M HCl using 300 mg l^−1^ of EAAP extract at 298 K. EAAP extract is shown to be a physisorbed process based on activation energies computed. The adsorption of EAAP extract over the electrode surface was discovered to be governed by the Langmuir adsorption isotherm. The huge capacitive loop in Nyquist plots reflects the adsorption of EAAP extract molecules on carbon steel samples.
